# Assessment of Expression Cassettes and Culture Media for Different *Escherichia coli* Strains to Produce Astaxanthin

**DOI:** 10.1007/s13659-018-0172-z

**Published:** 2018-06-06

**Authors:** Shun Li, Jun-Chao Huang

**Affiliations:** 1 Department of Economic Plants and Biotechnology, Yunnan Key Laboratory for Wild Plant Resources, Kunming Institute of Botany, Chinese Academy of Sciences, Kunming, 650201 People’s Republic of China; 2 University of Chinese Academy of Sciences, Beijing, 100049 People’s Republic of China

**Keywords:** Astaxanthin, *Escherichia coli*, Bacterial strains, Codon usage, Medium

## Abstract

**Abstract:**

Astaxanthin is a value-added ketocarotenoid with great potential in nutraceutical and pharmaceutical industries. Genetic engineering of heterologous hosts for astaxanthin production has attracted great attention. In this study, we assessed some key factors, including codon usage of the expressed genes, types of promoters, bacterial strains, and culture media, for engineered *Escherichia coli* to produce astaxanthin. The effect of codon usage was shown to be related to the types of promoters. *E. coli* DH5α was superior to other strains for astaxanthin production. Different culture media greatly affected the contents and yields of astaxanthin in engineered *E. coli*. When the expression cassette containing *GadE* promoter and its driving genes, *HpCHY* and *CrBKT*, was inserted into the plasmid pACCAR16ΔcrtX and expressed in *E. coli* DH5α, the engineered strain was able to produce 4.30 ± 0.28 mg/g dry cell weight (DCW) or 24.16 ± 2.03 mg/L of astaxanthin, which was a sevenfold or 40-fold increase over the initial production of 0.62 ± 0.03 mg/g DCW or 0.61 ± 0.05 mg/L.

**Graphical Abstract:**

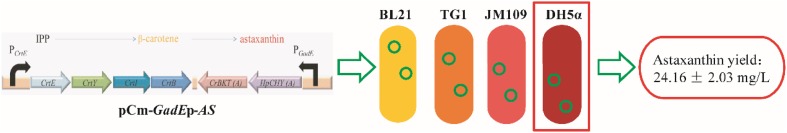

**Electronic supplementary material:**

The online version of this article (10.1007/s13659-018-0172-z) contains supplementary material, which is available to authorized users.

## Introduction

**Fig. 1:**
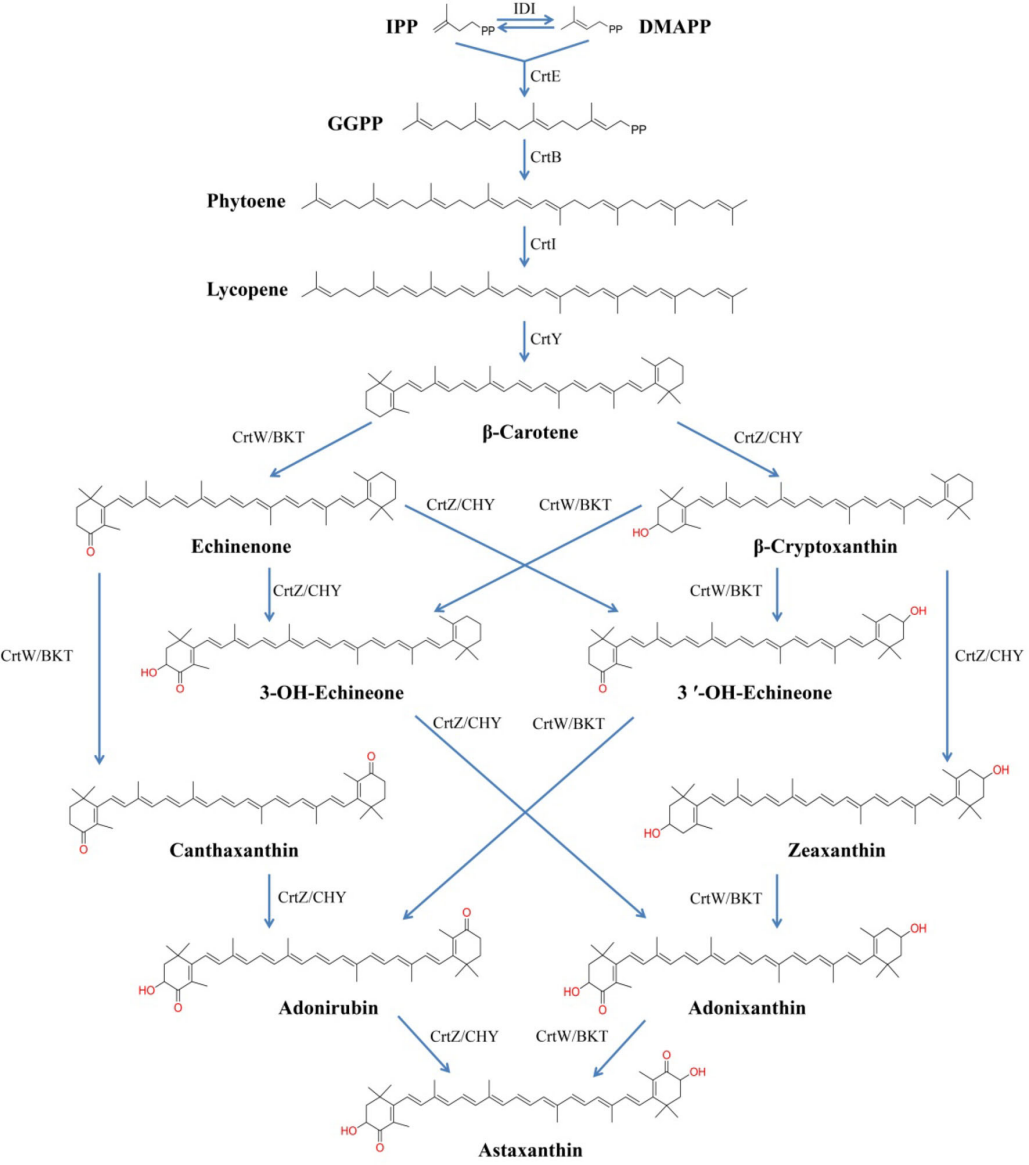
Biosynthetic pathways of astaxanthin. *IPP* isopentenyl pyrophosphate, *DMAPP* dimethylallyl diphosphate, *IDI* IPP isomerase, *CrtE* geranylgeranyl pyrophosphate synthase, *CrtB* phytoene synthase, *CrtI* phytoene desaturase, *CrtY* lycopene cyclase, *CrtW/BKT* β-carotene ketolase, *CrtZ/CHY* β-carotene hydroxylase

Astaxanthin (3,3′-dihydroxy-β,β-carotene-4,4′-dione) is a red ketocarotenoid with strong antioxidant activity, which has been widely applied in aquaculture, nutraceutical, pharmaceutical and cosmetic industries [1–3]. Currently, commercial natural astaxanthin is mainly extracted from the green alga *Haematococcus pluvialis* with relatively low productivity [4]. As a result, non-carotenogenic microorganisms, such as *Escherichia coli* [5–11] and *Saccharomyces cerevisiae* [12, 13], have been engineered to produce significant amounts of astaxanthin with different strategies, since the biosynthetic pathway of astaxanthin was elucidated at enzyme and gene levels [14]. Engineering an astaxanthin biosynthetic pathway in *E. coli* requires an exogenous carotenogenic pathway containing phytoene synthase (CrtB), phytoene desaturase (CrtI), lycopene cyclase (CrtY), β-carotene hydroxylase (CrtZ/CHY) and β-carotene ketolase (CrtW/BKT), (Fig. 1). Generally, two or more plasmids consisting of all the genes involved in converting isopentenyl pyrophosphate (IPP) to astaxanthin were co-transformed [6, 7, 9, 10] or the expression cassettes were integrated into the genomes of the targeted organisms [8, 11]. Strategies including combination of genes [6–8, 11] and their codon optimization [11], gene copy number adjustment [8, 9, 11], and so on, were integrated for improving astaxanthin production. Because these studies used different expression cassettes, strains, and culture conditions, it is not easy to assess the effects of these factors for the biosynthesis of astaxanthin in *E. coli*.

In this study we assessed the effects of codon usage, promoters, bacterial strains, and culture media on engineered *E. coli* for astaxanthin production. The CHY from *Haematococcus pluvialis* (HpCHY) and the BKT from *Chlamydomonas reinhardtii* (CrBKT) were adopted to convert β-carotene to astaxanthin based on our previous study [15]. We found bacterial strains and culture media were the most critical factors affecting the production of astaxanthin. When the astaxanthin pathway was constructed in one plasmid and expressed in *E. coli* DH5α strain and cultured with SBMSN medium containing 0.4% glucose, the engineered strain was able to produce 4.30 ± 0.28 mg/g DCW or 24.16 ± 2.03 mg/L of astaxanthin, which was a sevenfold or 40-fold increase over the initial production of 0.62 ± 0.03 mg/g DCW or 0.61 ± 0.05 mg/L.

## Results and Discussion

### Codon Usage of *HpCHY*-*CrBKT* Toward Astaxanthin Production

We previously found that the CHY from *Haematococcus pluvialis* (HpCHY) and the BKT from *Chlamydomonas reinhardtii* (CrBKT) were a better combination for efficiently converting β-carotene to astaxanthin in *E. coli* [15]. To investigate if codon optimization of the genes could improve astaxanthin production, *HpCHY* and *CrBKT* were synthesized based on the codon usage of *E. coli* [16]. The original algal *HpCHY*-*CrBKT* together with another codon optimizing ones based on *Arabidopsis thaliana* were used as controls. The three pairs of gene combinations were inserted into the plasmid pUC19 under the control of *lacZ* promoter. These constructs were expressed in β-carotene-producing *E. coli* JM109. The engineered bacteria cultured in Luria–Bertani (LB) medium with the inducer isopropyl β-D-thiogalactopyranoside (IPTG) demonstrated differential astaxanthin contents and productions (Tables 1, S1).

**Table 1:** Astaxanthin production of *E. coli* JM109 co-transformants (pACCAR16ΔcrtX+pUC-*lacZ*p-*HpCHY*-*CrBKT*) cultivated in LB medium with IPTG

Plasmid	OD600	Content (mg/g DCW)	Concentration (mg/L)	Ratio (%)
pACCAR16ΔcrtX+pUC-*lacZ*p-*HpCHY*-*CrBKT*	3.085 ± 0.428	0.374 ± 0.028	0.429 ± 0.028	34.247
pACCAR16ΔcrtX+pUC-*lacZ*p-*HpCHY*-*CrBKT (E)*	2.590 ± 0.050	0.624 ± 0.032	0.608 ± 0.050	81.578
pACCAR16ΔcrtX+pUC-*lacZ*p-*HpCHY*-*CrBKT (A)*	3.240 ± 0.060	0.466 ± 0.006	0.567 ± 0.004	44.976

Each value is shown as mean ± standard deviation (n ≥ 3)

The bacterium expressing the pair of *HpCHY*-*CrBKT (E)* with *E. coli*-based codon usage exhibited best astaxanthin content (0.62 ± 0.03 mg/g DCW) and production (0.61 ± 0.05 mg/L), but with lower biomass, suggesting that effective expression of the astaxanthin pathway driven by the *lacZ* promoter was detrimental to cell growth. The *Arabidopsis thaliana* codon optimized genes *HpCHY*-*CrBKT (A)* led to 0.47 ± 0.01 mg/g DCW or 0.57 ± 0.004 mg/L of astaxanthin, which is higher than that of the original algal genes *HpCHY*-*CrBKT*. These results showed that optimization of codon usage of the expressed genes could benefit astaxanthin production when the genes were driven by the ITPG-induced *lacZ* promoter.

### Assessment of *GadE* Promoter for Driving the Expression of *HpCHY*-*CrBKT*

GadE is a transcriptional activator of acid resistance system [17, 18]. GadE promoter is a stress-response one that was previously shown to improve the final titers of desired products [19]. To achieve the goal of producing high-levels of astaxanthin in the absence of inducer, the *GadE* promoter in the regulon was cloned 5′ of *HpCHY*-*CrBKT* and the resulting constructs were introduced into the β-carotene-producing *E. coli* JM109 strain.

**Fig. 2:**
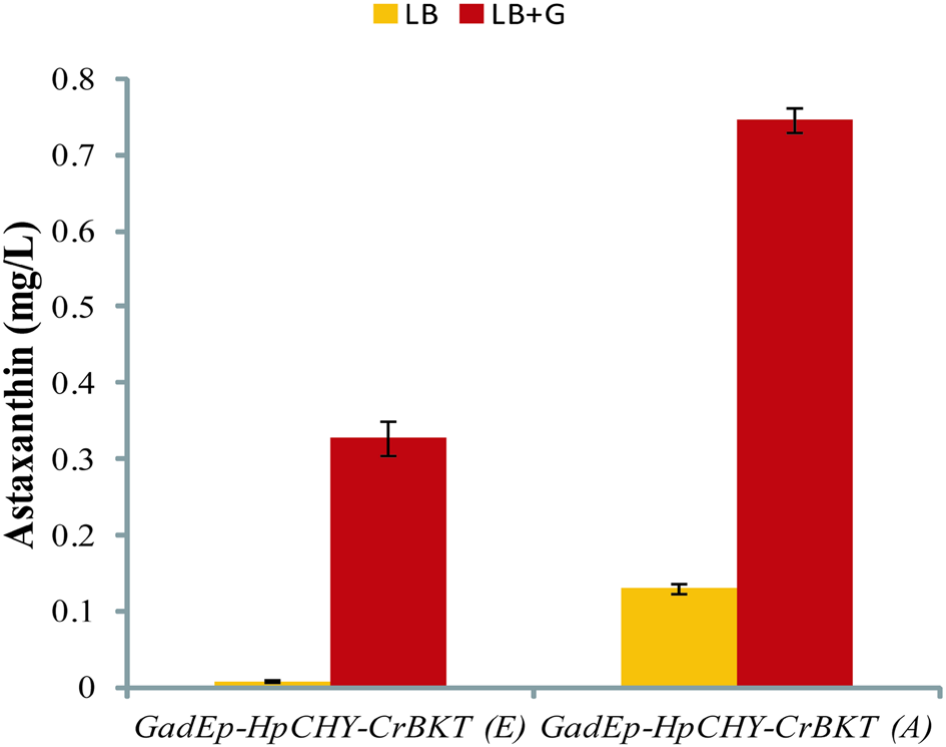
Astaxanthin production of *E. coli* JM109 co-transformants (pACCAR16ΔcrtX+pUC-*GadE*p-*HpCHY*-*CrBKT (E)*/*(A)*) cultivated in LB with or without glucose induction. Data represent averages from three or more replicate cultures; error bars show standard deviation

Interestingly, when driven by *GadE* promoter, the *HpCHY*-*CrBKT (A)* demonstrated better effects on astaxanthin production than the *HpCHY*-*CrBKT (E)* (Fig. 2 and Table S2). Furthermore, glucose greatly enhanced astaxanthin production, supporting that the expression of *GadE* was influenced by glucose [20]. The expression of *GadE* gene was affected by several different environmental cues, including transitions into stationary-phase, concentrations of sodium and magnesium [21], as well as exponential growth in minimal medium adjusted to pH 7 or below [18]. Thus, it is necessary to compare the performance of the astaxanthin pathway driven by *GadE* promoter in various *E. coli* strains and culture media.

### Assessment of *E. coli* Strains for Astaxanthin Production

**Fig. 3:**
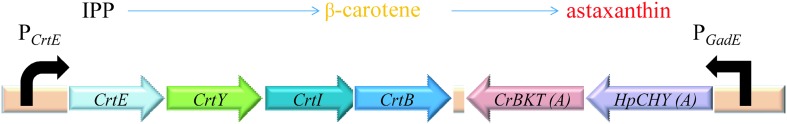
Schematic diagram of the plasmid pCm-*GadE*p-*AS*

A low-copy-number pACYC-derived plasmid was previously shown to be superior to a pUC-derived plasmid for the production of zeaxanthin in *E. coli* [22]. In addition, multi-plasmid transformation generally decreased the final production [22]. To alleviate cellular burden, we constructed a plasmid contained the whole astaxanthin pathway by inserting the expression cassette of the *HpCHY*-*CrBKT (A)* driven by *GadE* promoter into the plasmid pACCAR16ΔcrtX (Fig. 3).

**Fig. 4:**
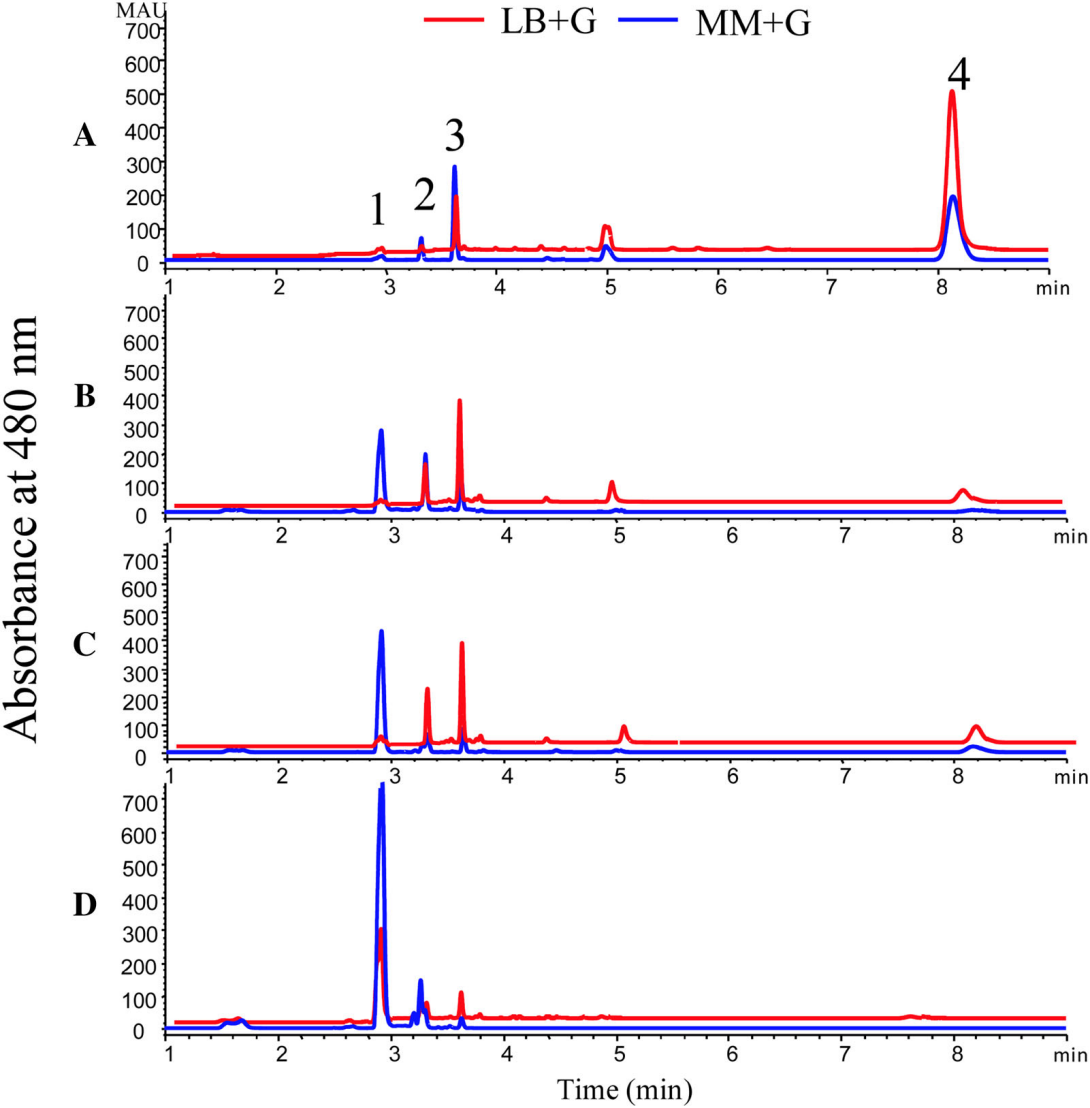
UHPLC analysis of carotenoids in different *E. coli* strains harboring plasmid pCm-*GadE*p-*AS*. *A* BL21; *B* TG1; *C* JM109; *D* DH5a. *1* astaxanthin; *2* adonixanthin; *3* canthaxanthin; *4* β-carotene

Previously, heterologous formation of carotenoids was shown to be related to *E. coli* strains [23]. Thus, the resulting construct was introduced into four *E. coli* strains so as to assess the relationship between strains and astaxanthin production. When cultured in LB+G medium (LB medium with 0.2% glucose) and MM+G medium (minimal medium with 0.6% glucose), the strains demonstrated various efficiency of astaxanthin biosynthesis (Fig. 4). Of the four strains, DH5α exhibited the best one for astaxanthin production in the context of contents, yields, and percentage (Fig. 4d). Compared with DH5α, the strains JM109 and TG1 shared similar carotenoid profiles in MM+G medium, but different in LB+G medium. The strains DH5α, JM109 and TG1 are derivatives of *E. coli* K-12, while they may have different precursor pools or different cellular environments for functional assembly of enzymes [23], or different membrane storage capacity for astaxanthin [5, 22], which may resulted in different productivity of astaxanthin. Furthermore, multi-omics analysis showed that, the strain DH5α was predicted to be the best producer for most constructs because of its higher expression of the biosynthetic pathways for tyrosine and phenylalanine [24].

In contrast, BL21 (DE3) showed the worst one for astaxanthin production due to the accumulation of intermediates, β-carotene and canthaxanthin (Fig. 4a). The genome of the strain BL21 lacks *rcsB* and *dsrA* genes, which are involved in the activation of *GadE* [25], possibly leading to the lower expression of *HpCHY*-*CrBKT (A)*. In addition, the accumulation of di-keto carotenoid canthaxanthin indicated that CrBKT had higher affinity to β-carotene than HpCHY.

### Assessment of Culture Media for Astaxanthin Production

**Fig. 5:**
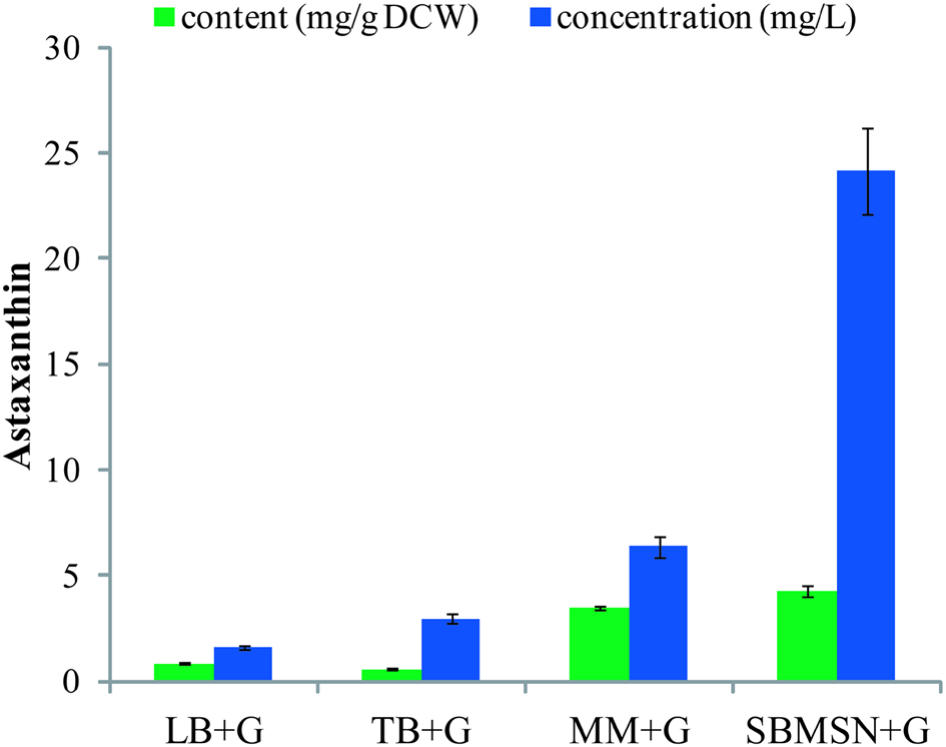
Astaxanthin production of *E. coli* DH5α transformants (pCm-GadEp-AS) cultivated in different media. Data represent averages from three or more replicate cultures; error bars show standard deviation

The popular complex LB medium allows the growth of *E. coli* up to a cell density of 1 g/L DCW, whereas a defined medium that contains the maximum non-inhibitive concentration of nutrients up to 15 g/L [26]. Therefore we cultivated the astaxanthin-producing DH5α cells in LB, TB, MM, and SBMSN media to investigate their effects on astaxanthin accumulation. The best concentrations of glucose were set to be 0.2% for LB and TB media, 0.6 and 0.4% for MM and SBMSN, respectively based on a pilot test (Table S3). The highest production of astaxanthin was achieved by the cells cultured in SBMSN+G medium, which reached 4.30 ± 0.28 mg/g DCW or 24.16 ± 2.03 mg/L (Fig. 5 and Table S4). The cells cultured in MM+G medium produced a similar content of astaxanthin (3.49 ± 0.09 mg/g DCW) to that in SBMSN+G, but a much lower yield of astaxanthin (6.39 ± 0.51 mg/L) due to its low biomass. SBMSN is a nutrient-rich and expensive medium containing high amounts of tryptone and yeast extract. In contrast, minimal medium is a defined and cheaper one. Hence, it is possible to achieve a much higher yield of astaxanthin by cultivating the cells in MM medium with further optimization of culture conditions, e.g., adjusting C:N ratios and adding Mg^2+^, Fe^2+^, Cu^2+^ to stimulate the activity of key enzymes.

In summary, we have assessed the most critical factors involved in astaxanthin biosynthesis in *E. coli*. By using the glucose-inducing *GadE* promoter to drive the codon optimized *CrBKT*-*HpCHY (A)* genes and constructing the whole astaxanthin pathway in a low copy number plasmid, a 40-fold increase of astaxanthin production was achieved by *E. coli* DH5α cells cultivated in the SBMSN+G medium. This study provides insight into the construction of much more robust strains for astaxanthin production in the future.

## Experimental Section

### Bacterial Strains, Culture Media and Plasmid

*Escherichia coli* JM109 [*endA1*, *gyrA96*, *hsdR17* (r_k_^−^, m_k_^+^), *recA1*, *relA1*, *supE44*, *thi*-*1*, Δ (*lac*-*proAB*), F′ (*traD36*, *proAB*^+^, *lacI*^q^ *lacZ*ΔM15)] was used for DNA mainipulations and carotenoids production. *E. coli* strains TG1 [Δ(*hsdMS*-*mcrB*)5, Δ(*lac*-*proAB*), *supE*, *thi*-*1*, F′ (*traD36*, *proAB*^+^, *lacI*^q^ *lacZ*ΔM15)], DH5α [*deoR*, *endA1*, *gyrA96*, *hsdR17* (r_k_^−^, m_k_^+^)), *recA1*, *relA1*, *supE44*, *thi*-*1*, Δ (*lac*)*U169* (ϕ80 *lacZ*ΔM15), F^−^], and BL21 [*E. coli* B, F^−^, *dcm*, *ompT*, *hsdS* (rB^−^ mB^−^), *gal*, λ (DE3)] were also used for carotenoids production. The carotenogenic plasmid pACCAR16ΔcrtX was used for *E. coli* to produce β-carotene [14].

The LB medium (pH 7.0) contains 10 g/L tryptone, 5 g/L yeast extract and 10 g/L NaCl. The TB medium (pH 7.0) contains 12 g/L tryptone, 24 g/L yeast extract, 4 g/L glycerol, 2.31 g/L KH_2_PO_4_ and 16.41 g/L K_2_HPO_4_·3H_2_O. The minimal medium (pH 7.0) used had the following composition: 2 g/L Glucose, 13.3 g/L KH_2_PO_4_, 4 g/L (NH_4_)_2_HPO_4_, 1.2 g/L MgCl_2_·6H_2_O, 1.86 g/L citric acid monohydrate, 10 mL 100 × Trace. The SBMSN (pH 7.0) contains 12 g/L tryptone, 24 g/L yeast extract, 1.7 g/L KH_2_PO_4_, 14.96 g/L K_2_HPO_4_·3H_2_O, 1 g/L MgCl_2_·6H_2_O, 1.42 g/L ammonium oxalate, and 2 g/L Tween-80.

### Genetic Methods

The genes *CHY* from *Haematococcus pluvialis* (*HpCHY*) and *BKT* from *Chlamydomonas reinhardtii* (*CrBKT*) were synthesized and cloned into pUC19 (SalI-BamHI) by Shanghai Generay Biotech according to our previous study [15] and “31 codon folding optimization” method reported by Boël et al. [16] for coexpression and codon optimization of the two genes.

The *GadE* promoter was amplified by PCR using genomic DNA of JM109 as the template and the primers GadEp F and R (Table S5). The product was inserted between HindIII and SalI of pUC19. Then the *GadE* promoter was cloned 5′of *HpCHY*-*CrBKT*, resulting in plasmids pUC-*GadE*p-*HpCHY*-*CrBKT (E)* and pUC-*GadE*p-*HpCHY*-*CrBKT (A)*.

The plasmid pCm-*GadE*p-*AS* was constructed by inserting the fragment *GadE*p-*HpCHY*-*CrBKT (A)* into the pACCAR16ΔcrtX using PCR-based and seamless connection approaches.

PCR amplifications were performed using Prime STAR Max premix (Takara) and a thermal cycler (Eppendorf). Restriction enzymes were purchased from New England BioLabs. Hieff Clone™ Plus One Step Cloning Kit was purchased from Shanghai Yeasen Biotech. All the primers used in this study were listed in Table S5.

### Growth Condition of Astaxanthin Production

Single colonies were picked from the plates and inoculated into 12 mL tubes containing 3 mL LB medium. Chloramphenicol (34 mg/L) and ampicillin (100 mg/L) were added to the culture medium as required. The culture was grown to OD600 = 2.0 at 37 °C in a rotary shaking incubator set to 220 rpm. Then, 210 µL of seed-culture was inoculated into new 12 mL tubes containing 3 mL medium, with appropriate antibiotics and grown at 28 °C, 220 rpm for 48 h [22]. For *lacZ* promoter to drive gene expression, 1 mM (final concentration) of IPTG was added at OD600 = 1. After 48 h growth, cells were collected for measurement of carotenoids production. Cell growth was measured at the optical density of OD600 nm and the cell density was converted into DCW (g/L) using standard curves (Fig. S1).

### Extraction and Analysis of Carotenoids

Carotenoids analysis was performed as described previously [27]. Briefly, 1 mL cells were harvested by centrifugation and the cell pellets were used for the extraction of pigments. The cells were extracted with acetone until the color of the sample faded.

The extracts were filtered through a 0.22 μm Millipore organic membrane, and then analyzed by an Agilent Ultra High Performance Liquid Chromatography (UHPLC) 1290 Infinity, which is equipped with an Agilent Eclipse plus C18 RRHD 1.8 μm column (2.1 × 50 mm). The mobile phase consisted of elution A (20% water, 60% acetonitrile, 5% isopropanol and 15% methanol) and elution B (80% acetonitrile, 5% isopropanol and 15% methanol). The extracts were eluted at a flow rate of 0.5 mL/min with following process: 100% A for 1 min, and then a liner gradient from 100% A to 100% B within 1 min, followed by 100% B for 8 min. Individual pigment was identified by comparing the retention times and absorption spectra to standard carotenoids, and the concentrations of pigments were determined by standard curve and peak areas (Fig. S2).

## Electronic supplementary material

Below is the link to the electronic supplementary material.

Supplementary material 1 (DOCX 107040 kb)
