# Supplementary material for: Assessment of Expression Cassettes and Culture Media for Different Escherichia coli Strains to Produce Astaxanthin
Source: Nat Prod Bioprospect. 2018 Jun 6;8(5):397–403. doi: 10.1007/s13659-018-0172-z (PMC6109440; doi:10.1007/s13659-018-0172-z)
Supplement: Supplementary file 1 — Supplementary material 1 (DOCX 107040 kb) [file 13659_2018_172_MOESM1_ESM.docx]

**Supporting Information**

**Table S1** Astaxanthin production of *E. coli* JM109 co-transformants (pACCAR16ΔcrtX+pUC-*lacZ*p-*HpCHY*-*CrBKT*) cultivated in LB medium with IPTG.

**Table S2** Astaxanthin production of *E. coli* JM109 co-transformants (pACCAR16ΔcrtX+pUC-*GadE*p-*HpCHY*-*CrBKT (E)*/*(A)*) cultivated in LB with or without glucose induction.

**Table S3** Astaxanthin production of *E. coli* DH5α transformants (pCm-GadEp-AS) cultivated in different media with different concentration of glucose.

**Table S4** Astaxanthin production of *E. coli* DH5α transformants (pCm-GadEp-AS) cultivated in different media.

**Table S5** Primes used in this study.

HindIII, SalI sites are underlined.

**Fig. S1** The standard curve of OD600-dry cell weight (DCW) of *E. coli* JM109 and DH5α.

**Fig. S2** The standard curve of astaxanthin concentration-peak areas.
